# Factors affecting plasmid production in *Escherichia coli *from a resource allocation standpoint

**DOI:** 10.1186/1475-2859-8-27

**Published:** 2009-05-22

**Authors:** Drew S Cunningham, Richard R Koepsel, Mohammad M Ataai, Michael M Domach

**Affiliations:** 1 Department of Chemical Engineering, Carnegie Mellon University, Pittsburgh, Pennsylvania, USA; 2 Department of Chemical and Petroleum Engineering, University of Pittsburgh, Pittsburgh, Pennsylvania, USA

## Abstract

**Background:**

Plasmids are being reconsidered as viable vector alternatives to viruses for gene therapies and vaccines because they are safer, non-toxic, and simpler to produce. Accordingly, there has been renewed interest in the production of plasmid DNA itself as the therapeutic end-product of a bioprocess. Improvement to the best current yields and productivities of such emerging processes would help ensure economic feasibility on the industrial scale. Our goal, therefore, was to develop a stoichiometric model of *Escherichia coli *metabolism in order to (1) determine its maximum theoretical plasmid-producing capacity, and to (2) identify factors that significantly impact plasmid production.

**Results:**

Such a model was developed for the production of a high copy plasmid under conditions of batch aerobic growth on glucose minimal medium. The objective of the model was to maximize plasmid production. By employing certain constraints and examining the resulting flux distributions, several factors were determined that significantly impact plasmid yield. Acetate production and constitutive expression of the plasmid's antibiotic resistance marker exert negative effects, while low pyruvate kinase (Pyk) flux and the generation of NADPH by transhydrogenase activity offer positive effects. The highest theoretical yield (592 mg/g) resulted under conditions of no marker or acetate production, nil Pyk flux, and the maximum allowable transhydrogenase activity. For comparison, when these four fluxes were constrained to wild-type values, yields on the order of tens of mg/g resulted, which are on par with the best experimental yields reported to date.

**Conclusion:**

These results suggest that specific plasmid yields can theoretically reach 12 times their current experimental maximum (51 mg/g). Moreover, they imply that abolishing Pyk activity and/or transhydrogenase up-regulation would be useful strategies to implement when designing host strains for plasmid production; mutations that reduce acetate production would also be advantageous. The results further suggest that using some other means for plasmid selection than antibiotic resistance, or at least weakening the marker's expression, would be beneficial because it would allow more precursor metabolites, energy, and reducing power to be put toward plasmid production. Thus far, the impact of eliminating Pyk activity has been explored experimentally, with significantly higher plasmid yields resulting.

## Background

Microorganisms such as *Escherichia coli *are routinely used for producing recombinant proteins. The recombinant gene of interest is usually carried on a high copy number plasmid in order to boost gene dosage and, ultimately, process yield. While the usual view of the plasmid's role is simply as the vehicle for recombinant protein expression, the appeal of plasmids as gene therapy vectors has recently sparked interest in the production of plasmid DNA itself as the process end-product. Once thought inferior to viral vectors, plasmids are being reconsidered as viable vector alternatives for gene therapies and vaccines because they are safer, non-toxic, simpler to produce, and can accommodate larger inserts. In fact, of the 261 gene therapy clinical trials approved from 2006–08, 22% employed a plasmid DNA vector [1].

To date, efforts aimed at achieving high upstream plasmid yields have focused primarily on the optimization of fermentation conditions (e.g. medium design, cultivation mode, feeding strategy, pH or DO control), and/or on the usage of a plasmid containing a high copy number, temperature-sensitive origin of replication (*ori*) [2-8]. Designed media typically contain several or all amino acids and/or nucleosides in addition to minimal salts and a carbon source such as glycerol, which confers a slow growth rate [4-7]. Reduced growth rate, in turn, has been linked to elevated copy numbers [9]. In *E. coli*, pUC-based plasmids are the norm. A mutation in its RNA primer and elimination of the *rop *(repressor of primer) gene result in much higher copy numbers than those observed for its originator, pBR322 [10]. Moreover, its high copy phenotype can be bolstered several-fold further upon temperature-shift (e.g. ≤ 37°C to 42°C) [2,5,7,8,10,11]. The best yield reported thus far employing combinations of such techniques has been 51 mg/g [8], and the highest productivity has been 36 mg/L/h [5].

Unlike other therapeutic products such as streptokinase, vaccines must be produced and administered in population- as opposed to subpopulation-scale quantities. For 100 million doses at 1 mg per dose [11], and 10 different vaccine products, about 100 million liter-hours of productive capacity would now be required. Clearly at such a scale, a 10-fold reduction or more in required capacity would favorably impact the economics. Additionally, it would be less challenging to coordinate the production of multiple vaccine products. Apart from scale, the US FDA currently recommends that, in addition to proteins, RNA, and cell envelope components, both genomic DNA and non-supercoiled plasmid isoforms should be considered as contaminants [12,13]. Thus, commencing purification with a higher relative abundance of efficacious material would lessen the impact of downstream losses and lower the contaminant level per mass of isolated product. The considerations of scale and purity indicate that it would be useful to determine what the maximum theoretical plasmid yield is for a given set of nutritional conditions, and compare that to current yields.

To estimate what the maximum yield might be for aerobic growth on glucose minimal medium, a simple carbon balance can be performed. The yield determined in such a manner will not, however, consider any constraints that can arise when nested metabolic networks traffic carbon from inputs (glucose, CO_2_) to products (biomass, CO_2_, acid by-products, plasmid, antibiotic resistance marker for plasmid selection). This simple calculation for a model pUC-based plasmid (pGFPuv, 3.3 kb) shows that yields of 742 mg/g (33,000 copies/cell) might be attained when 30% of glucose carbon is released as CO_2 _and there is no marker or acetate production (see Appendix). This theoretical yield is 15-fold greater than the best that has been achieved experimentally to date [8]. Because the currently employed strategies do not approach the estimated theoretical yield, a metabolic flux analysis might help elucidate why this is the case. Such an analysis would reveal whether constraints arise and/or suboptimal metabolic trafficking detracts from the observed value of the yield, given that the negative control over replication is not limiting.

Our goal, therefore, was to develop a stoichiometric model of *E. coli *metabolism for batch aerobic growth on glucose minimal medium, with the model's objective being to maximize plasmid production. Such models have been developed before for *E. coli *and other microorganisms, like *Bacillus subtilis*, to assess the production capacity for amino acids and nucleotides by *E. coli *[14] or folic acid by *B. subtilis *[15]. Not only do such models allow for a more appropriate determination of the maximum theoretical yield for a given set of conditions, but they also reveal how carbon should traffic through the metabolic network to achieve the ultimate yield. This information is particularly useful because these optimal carbon flux distributions can be examined to rationally identify gene candidates whose mutation or up/down-regulation might positively impact product yield. Thus, results from this study could serve as guides for engineering host strains for plasmid production.

Using flux analysis, we determined the factors that significantly impact plasmid production, the optimal flux distribution, and the theoretical maximum yield of plasmid production. This paper describes the key factors found. First, as one would expect, both the production of acetate and the constitutive expression of the plasmid's antibiotic resistance marker exert negative effects. Interestingly, nil pyruvate kinase (Pyk) flux and the generation of NADPH by nicotinamide nucleotide transhydrogenase activity were found to offer yield-enhancing effects. The highest theoretical yield (592 mg/g) resulted when the optimal flux distribution showed no marker or acetate production, nil Pyk flux, and the maximum allowable transhydrogenase activity. These results suggest that specific plasmid yields can theoretically reach 12 times their current experimental maximum. Moreover, they imply that Pyk deletion and/or transhydrogenase up-regulation would be useful strategies to implement when designing host strains for plasmid production. After presenting these results, we describe the results of published experiments [16] that were aimed at pursuing some of the findings from the analysis. The experimental results indicate that one advantageous mutation identified by the analysis, eliminating Pyk activity, does indeed increase plasmid titer.

## Methods

### Metabolic Reaction Network

**Figure 1:**
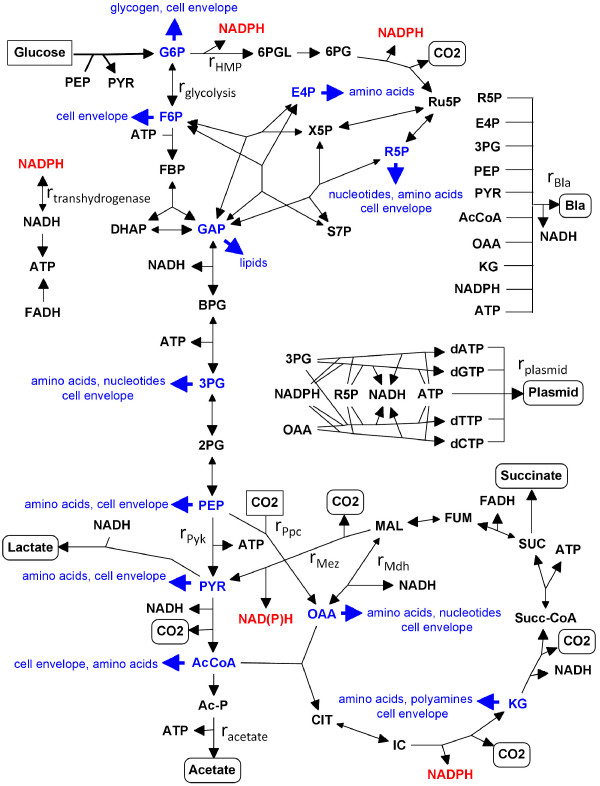
**Metabolic reaction network of plasmid production in *E. coli *for aerobic growth on glucose minimal medium**. Points of carbon entry are boxed. Points of carbon exit are circled except for drains on precursor metabolites for biomass synthesis, which are denoted in blue and by large shaded arrowheads. Double-ended arrows represent potentially reversible reactions, with the larger arrowhead depicting the net direction. Key fluxes discussed in the text are labeled as *r*_*i*_. Specific details on reaction stoichiometries can be found in the Methods, and abbreviations are defined in the Abbreviations section.

Figure 1 shows the metabolic network that was subjected to analysis. Glycolysis, the hexose monophosphate (HMP) pathway, and the tricarboxylic acid (TCA) cycle comprise the bulk of the reactions. Others include the uptake of glucose via the phosphotransferase system (PTS), the potential generation of acid by-products (acetate, lactate, and succinate), and two anaplerotic reactions (phosphoenolpyruvate carboxylase, Ppc; malic enzyme, Mez). Known biochemistry was used to establish the stoichiometry and directionality of these reactions [17,18]. Biomass is produced from precursor metabolites (Fig. 1, *large blue arrows*) according to the composition specified by Neidhardt et al. [17]. Because the goal of this work was to determine the optimum flux distribution for maximum plasmid production, reactions were included for the synthesis of deoxyribonucleoside triphosphate (dNTP) building blocks from precursor metabolites, the synthesis of the model plasmid (pGFPuv; Clontech, GenBank #U62636) from the appropriate numbers of dNTPs, and for the constitutive expression of the antibiotic resistance marker contained on that plasmid (i.e. β-lactamase; Bla). Assuming that 1.36 mol ATP are required per mol dNTP for polymerization [17], the plasmid reaction is:

(1)

The stoichiometry used for each of the four dNTPs from precursors was that of Neidhardt et al. [17], with the demand for single carbon units satisfied by the synthesis of serine as described by the authors. The overall stoichiometry of the Bla reaction was established using its amino acid composition, 4.324 mol ATP/mol amino acid for polymerization, and accounting for single carbon units as serine [17]. The net reaction from precursors is as follows:

(2)

The production of biomass, plasmid, and Bla require energy and reducing power. Aside from substrate-level phosphorylation, ATP can be produced via electron transport and oxidative phosphorylation. For P/O ratios, we assumed that 1 mol FADH_2 _generates 1 mol ATP, and 1 mol NADH generates 2 mol ATP. NADH and NADPH are produced by the central metabolic pathways, and the model also contains a reversible transhydrogenase reaction that allows for their interconversion.

### Model Definition and Solution Method

At steady state (i.e. balanced exponential growth), the *m *reactions and *n *metabolites of a given reaction network collectively define a set of linear mass balance equations that can be formulated as:

(3)

where ***S ***is the *n *× *m *stoichiometric matrix, ***r ***is an *m *× 1 vector of the unknown metabolic reaction rates or fluxes, and ***b ***is an *n *× 1 vector of the rates of metabolite consumption or generation.

The objective function for *all *optimizations carried out in this work was to maximize plasmid production:

(4)

The mass balances from Equation (3) were employed as constraints, and bounds were imposed on irreversible fluxes to ensure that their values remained non-negative. The drain on a given precursor metabolite for biomass synthesis (Fig. 1, large blue arrows) was specified as an equality constraint by using the biomass composition of Neidhardt et al. [17] and multiplying its amount (mmol/g) by an assumed specific growth rate (*μ*) of 0.74 h^-l^. The amount of NADPH needed for biomass synthesis was fixed at 18.225 *μ *mmol/g/h, while the required ATP was defined as a greater-than-or-equal-to constraint (41.223 *μ *mmol/g/h) [17]. With the constraint formulated in this manner, the model is guaranteed to generate *at least *the amount of energy needed to produce macromolecular building blocks from precursor metabolites as well as that required for polymerization.

Carbon is supplied to the network exclusively from glucose or CO_2 _(Fig. 1, *boxed*). Glucose uptake was limited to an upper bound based on a typical biomass yield of 0.35 g/g glucose for aerobic growth in minimal medium containing glucose concentrations that are high enough to permit acetate excretion [19]. Because the objective was always to maximize plasmid production, this upper bound was adopted by every model posed in this study in order to supply the maximum allowable carbon to the system. Further, it agrees well with reported experimental glucose uptake under the growth conditions considered [20]. In addition to biomass, carbon was permitted to be extracted from the network as CO_2_, acid by-products, plasmid, and/or Bla (Fig. 1, *circled*).

Bla production was linearly coupled to plasmid synthesis as follows:

(5)

where *α*, whose units are mol Bla/mol plasmid, represents the product of transcriptional (number of transcripts per gene) and translational (number of ribosomes per transcript in a polysome) activities. Thus, it reflects the bilinear combination of promoter strength and ribosome binding/content. To put *α *into a quantitative perspective, consider its value for fully activated LacZ production. A value of 1838 (about 56 transcripts per gene and about 32 ribosomes per transcript) was determined in a modeling study [21], which was reported to be in good agreement with experimental data [22]. Variation of *α *and relaxation of Equation (5) to an inequality constraint based on the availability of protein synthesis machinery is described in the text for particular modeling aims. Other key fluxes that were either constrained or unconstrained in order to explore particular flux trafficking scenarios are *r*_*acetate*_, *r*_*Pyk*_, and *r*_*transhydrogenase *_(Fig. 1). The first two of these fluxes can be assumed to be unconstrained unless specified to the contrary, whereas *r*_*transhydrogenase *_was constrained to be zero unless otherwise noted.

To solve the optimization problems posed in this work, the model was constructed in GAMS, and the recursive mixed-integer linear programming algorithm described by Lee et al. [23] was employed.

## Results

### Optimal Flux Distribution for Maximum Plasmid Production

In order to determine the optimum flux distribution for maximum plasmid production, the model was solved for *α *= 0 in Equation (5). This *α*-value corresponds to a situation where nil antibiotic resistance marker production is allowed to occur. This case provides the upper bound value of the maximal yield, which represents the horizon for emerging selection techniques that do not rely on high levels of constitutive antibiotic resistance expression [24-26]. Later, the effect of nonzero *α *is considered.

**Figure 2:**
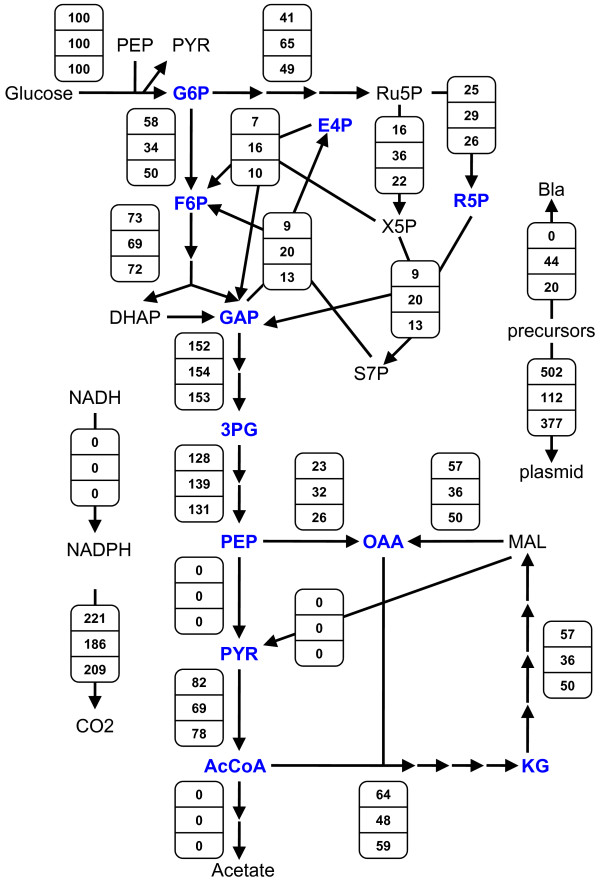
**Optimal carbon flux distributions for maximum plasmid production in *E. coli***. In all three distributions,*r*_*transhydrogenase *_was constrained to zero, while *r*_*acetate*_and *r*_*Pyk *_were not constrained. Distributions vary by their assigned value of *α *in Equation (5) and/or whether or not a limit was imposed on Bla production. *Top*:*α *= 0. *Middle*:*α *= 250; unlimited Bla production. *Bottom*:*α *= 250; Bla production limited to 20% of total protein (Equation (7), *f *= 0.20). For simplicity, the network in Fig. 1 has been condensed and fluxes (mmol/g/h) have been expressed as % of glucose uptake, except *r*_*Bla *_(expressed as % of total protein) and *r*_*plasmid *_(expressed as yield in mg/g). The production of lactate and succinate have also been omitted, as these fluxes were nil for all distributions.

Figure 2 (*top*) shows the optimal flux distribution. The maximum theoretical plasmid yield predicted is 502 mg/g. No input carbon is wasted in the formation of acidic by-products (i.e. acetate, lactate, or succinate); hence, all carbon not consumed for biomass synthesis is put towards plasmid production or released as CO_2_. Note that this yield does not perfectly match that predicted by the simple carbon balance (742 mg/g, Appendix). That number, however, was calculated assuming a value of 30% for the fraction of input carbon given off as CO_2_, whereas the model's stoichiometry provides a value of 36.8%. Interestingly, the optimal solution results when Pyk flux is nil. Since this flux is nonzero in wild-type *E. coli*, it may represent a site where targeted mutation could prove beneficial when designing host strains for maximal plasmid production. The impact of Pyk will be further examined below, and experimental results concerning Pyk-deficient *E. coli *will be described in the Discussion section.

### Effects of Antibiotic Resistance Marker Production

Because many current plasmids rely on the constitutive production of an antibiotic resistance marker for selection (e.g. Bla), some fraction of the available precursor metabolites, energy, and reducing power must be consumed to synthesize this protein. Therefore, the more marker produced, the less that these resources are available for plasmid production. Indeed, markers are usually produced at levels far exceeding those that are necessary for plasmid maintenance and selection, with levels at 20% of total cellular protein not uncommon in the case of high copy plasmids [27,28]. Aside from creating a metabolic burden, excess marker synthesis can substantially impact downstream purification due to the hyper-sensitivity of some patients to product contamination by marker protein [29].

**Figure 3:**
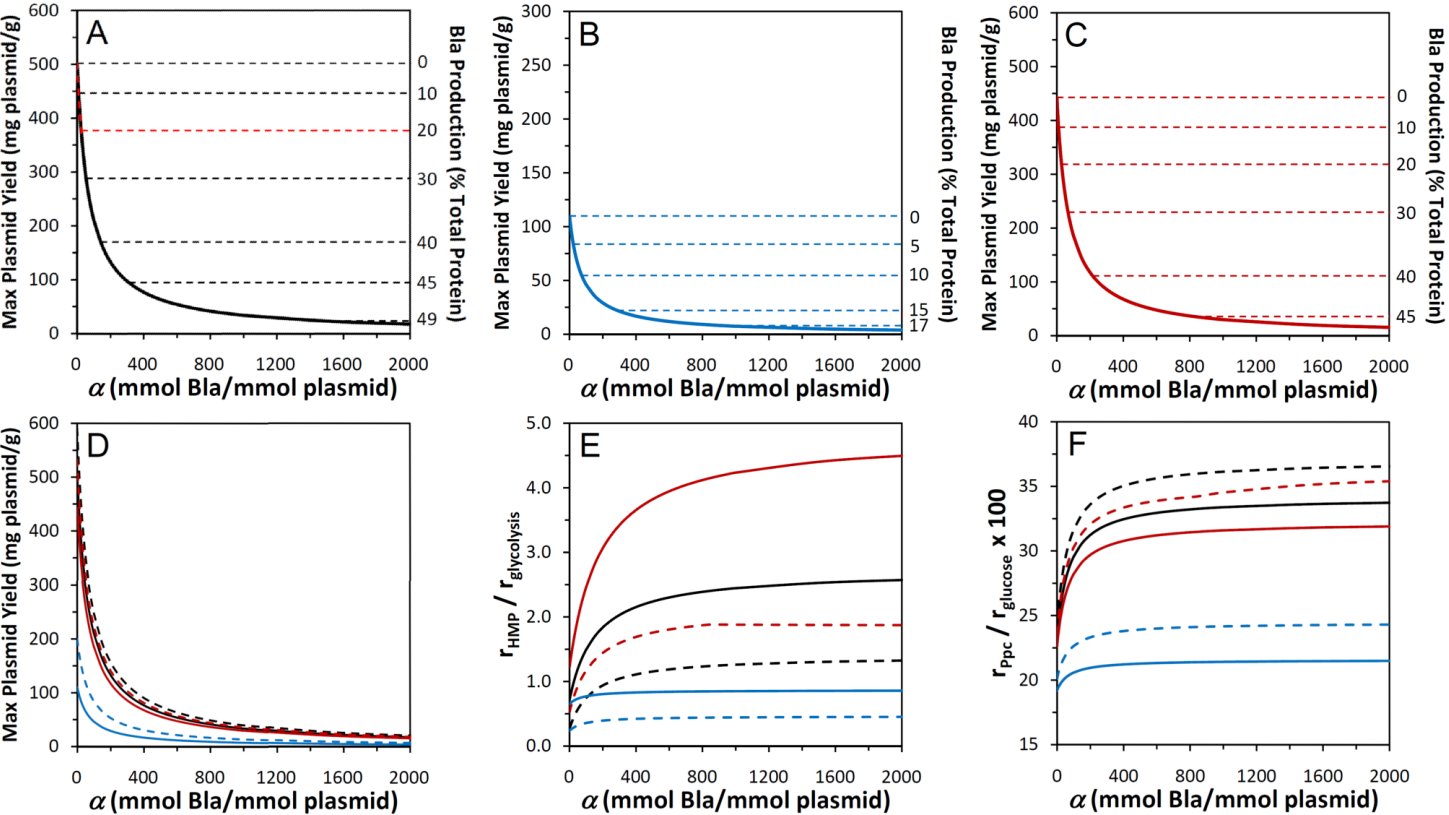
**Maximum plasmid yield and other key fluxes as a function of *α***. **(A-C) **Maximum plasmid yield for different *r*_*acetate *_and *r*_*Pyk *_scenarios, variable limits on Bla production, and *r*_*transhydrogenase *_= 0. The fluxes *r*_*acetate *_and *r*_*Pyk *_were: **(A) **unconstrained, but adopted values of zero, **(B) **set to JM101 (wild-type) values, or **(C) **fixed at PB25 (Pyk-deficient) values. *Solid lines*: No upper limit imposed on Bla production. *Dashed lines*: Bla production limited to selected percentages of total protein. Yield decreases as a function of promoter strength *α *along a *solid line *if Bla production is unconstrained. When Bla production is limited to a particular percentage, the yield decreases along a *solid line *until it becomes independent of *α *at its respective flat *dashed line*. **(D) **Effect of transhydrogenase activity on plasmid yield. *Solid lines*: Redrawn from (A-C) for comparison where unconstrained (*black*), PB25 (*red*), and JM101 (*blue*) values for *r*_*acetate *_and *r*_*Pyk *_are assumed, and *r*_*transhydrogenase *_= 0. *Dashed lines*:Increased maximum plasmid yield for unconstrained (*black*), PB25 (*red*), and JM101 (*blue*) cases when *r*_*transhydrogenase *_> 0 per Equation (10). **(E) **HMP:glycolysis flux ratio when *r*_*transhydrogenase *_= 0 (*solid lines*) or when *r*_*transhydrogenase *_> 0 per Equation (10) (*dashed lines*). Contrasted are the unconstrained (*black*), PB25 (*red*), and JM101 (*blue*) cases. **(F) **Ppc flux as % glucose uptake when *r*_*transhydrogenase *_= 0 (*solid lines*) or > 0 (*dashed lines*). Cases compared and color coding as in (D) and (E).

To gauge the extent to which concomitant marker production negatively impacts plasmid yield, the model was solved for many different values of *α *in Equation (5). Figure 3A (*black solid line*) shows how maximum plasmid yield varies with *α *and the corresponding amount of Bla produced (expressed as a percentage of total protein). As expected, as *α *and thus Bla production increase, the plasmid yield decreases. The decrease is remarkably steep for the lowest values of *α*. By *α *= 250, for example, the marker constitutes roughly 44% of total protein, and its drain on resources diminishes plasmid-producing capacity by over 75% (502 mg/g for *α *= 0 to 112 mg/g).

Figure 2 (*middle*) shows the flux distribution for this *α *= 250 case. This value of *α *corresponds to a situation where Bla's promoter strength is about one-third that of the *lac *promoter (23 transcripts per gene) [21,22], and the number of bound ribosomes per transcript is about 11 based on Bla's message length and a typical ribosome spacing distance [29]. As with the *α *= 0 case, no carbon is wasted in the form of acid by-products, and the Pyk flux is zero. In contrast to the *α *= 0 case, HMP and Ppc fluxes are greater, whereas glycolytic and TCA cycle fluxes are lessened in order to provide the appropriate balance of precursors, energy, and reducing power for *both *plasmid and Bla production (see also Fig. 3E & Fig. 3F).

### Effects of Imposing a Limit on Marker Production

Because the marker represents such a large fraction of total protein even at modest values of *α *(Fig. 3A), we also considered what would happen to plasmid production if the cell had an upper bound for marker production. This ceiling could be set by a limitation in the transcriptional and/or translational machinery, such as a limited availability of free ribosomes. If this limit existed and was reached, plasmid replication could continue while marker synthesis had hit its ceiling, a phenomenon which has been observed experimentally [30]. In this case, marker synthesis would no longer occur with linear dependence on plasmid synthesis as dictated by Equation (5), despite the fact that there will always be a one-for-one increase in marker genes with plasmids. Therefore, the ultimate burden that marker production imposes on plasmid yield would be lessened, and more resources would be available for use in plasmid production. Thus, this calculation allows for the maximal plasmid yield to be assigned a range, where a tight or decoupled relationship between gene dosage and marker level establishes a lower or upper bound, respectively.

Implementing such decoupling in the model is straightforward. When one is interested in limiting Bla production to some fraction (*f*) of total protein, the stoichiometries of protein biomass and Bla can be used to formulate an expression for *f*. For example, biomass contains 0.488 mmol/g of alanine, meaning that the flux of alanine is 0.488 *μ *mmol/g/h in our model, and one Bla contains 28 alanine residues. Therefore,

(6)

Depending on the specified values of *α *and *f *in a particular model, *r*_*Bla *_will be constrained by making either Equation (5) or (6) active. If *α *is small enough that Bla production does not reach the upper bound established by *f*, then Equation (5) is active. This leaves Bla production linearly proportional to plasmid production, meaning that by maximizing plasmid production, Bla production is concomitantly being maximized. If, on the other hand, *α *is large enough such that the limit imposed by *f *is hit, then Equation (6) is employed in lieu of Equation (5). That is,

(7)

where Equation (6) has been rearranged, and α* denotes the value of *α *where Bla production exactly equals *f*, which can be determined using the results in Fig. 3A (*black solid line*). Equation (7) makes Bla production a fixed drain on resources when *α *≥ α*, meaning that Bla is no longer being simultaneously maximized with plasmid production. Sub-maximal Bla production means that a greater amount of resources are available for plasmid production.

As an example, assume that the upper limit for Bla production is 20% of total protein (*f *= 0.20), a value that, as stated previously, is not uncommon for marker production when copy numbers are high [27,28]. According to Fig. 3A, when α* = 24, the 20% limit is reached. Hence, when modeling how the maximum plasmid yield varies with *α *for this case, Equation (7) can be employed with the appropriate α*. The result is shown in Fig. 3A (*red dashed line*), along with six other cases of *f *(*black dashed lines*). In each case, the maximum plasmid yield varies with *α *along the *solid line *until its respective value of α *. Thereafter, Bla production is at its imposed limit, and the maximum achievable plasmid yield becomes independent of *α*. In the *f *= 0.20 case, this means that *E. coli *can theoretically yield 377 mg/g of plasmid in addition to expressing the marker at 20% of total protein if *α *≥ α*.

Because Bla production becomes a fixed load for a given *f *when *α *≥ α*, the flux distribution for any *α *in that span is identical. Figure 2 (*bottom*) shows the *f *= 0.20 flux distribution when *α *= 250 for comparison to the previously described cases where no limit was imposed on Bla production and *α *= 0 (*top*) or *α *= 250 (*middle*). As mentioned previously, in the case of non-limited Bla production and *α *= 250, the attainable plasmid yield falls from its absolute maximum of 502 mg/g (*α *= 0) to 110 mg/g due to the large amount of concomitant marker synthesis (44% of total protein). When marker synthesis is instead limited to 20% of total protein, the increased availability of resources that would otherwise go to marker production are instead channelled into additional plasmid synthesis. This allows for a substantially higher maximum plasmid yield of 377 mg/g. Such an effect, as demonstrated here for increased plasmid yield with decreased marker production, has also been observed experimentally for recombinant protein production, where 2-fold greater yields were achieved when marker synthesis was significantly decreased by employing a weakened promoter [27].

Regarding key fluxes, as with the two previous cases, no acid by-products are formed, and there is nil Pyk flux (Fig. 2, *bottom*). In fact, unless constrained to be nonzero, these two fluxes always adopt a value of zero when the model's objective is to maximize plasmid production, regardless of the value of *α *and/or the imposition of an upper bound on Bla production.

### Effects of Pyruvate Kinase Flux and Acetate Production

**Figure 4:**
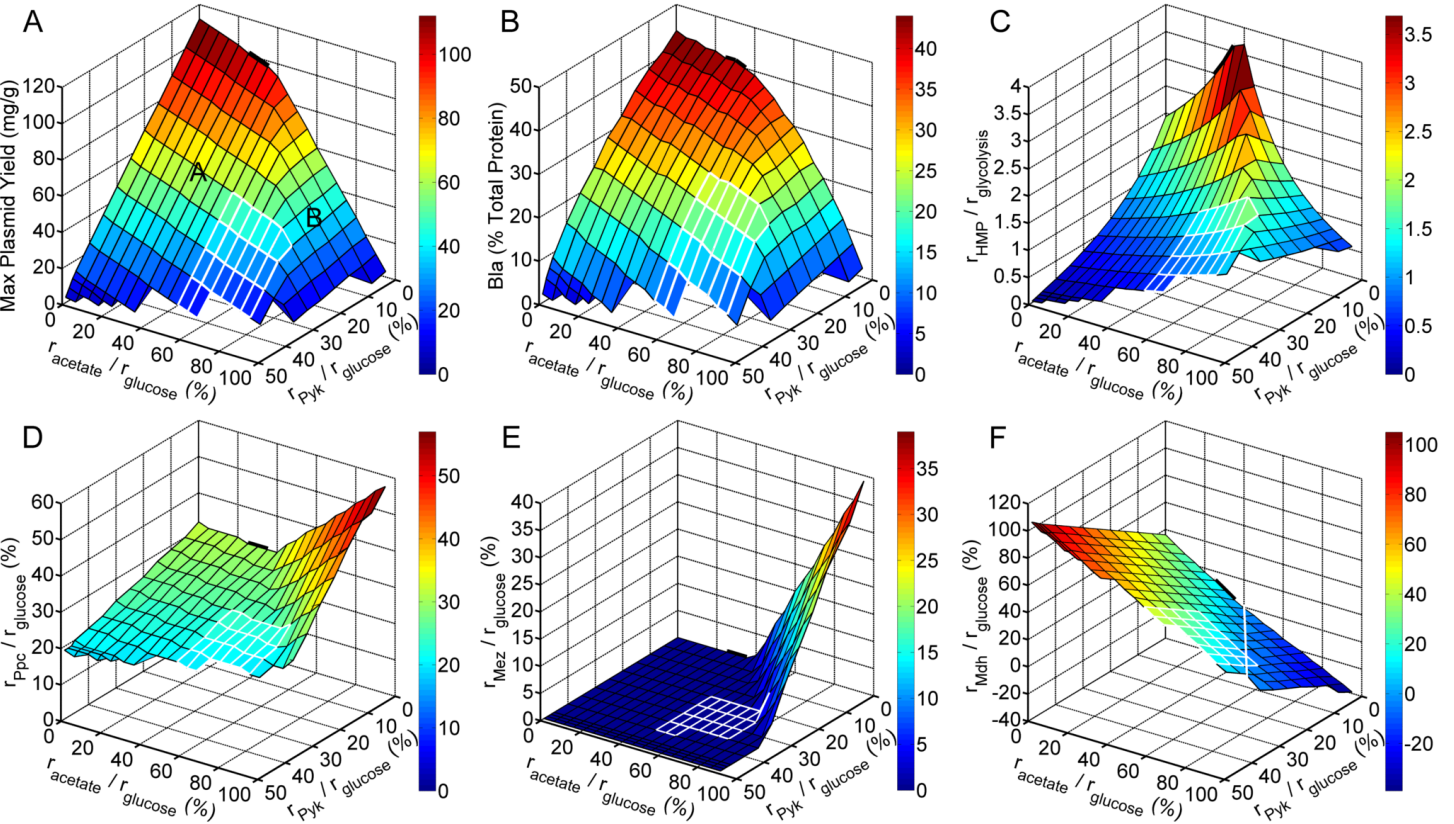
**Effects of Pyk flux and acetate by-production on maximum plasmid yield and other key fluxes**. The model was run for different constrained combinations of *r*_*acetate *_and *r*_*Pyk *_in Equations (8) and (9), with *α *= 250 in Equation (5) and *r*_*transhydrogenase *_= 0 in every case. Specific (*r*_*acetate*_, *r*_*Pyk*_) zones are drawn in each panel for JM101 (wild-type) and PB25 (*pykF pykA*) based on experimentally determined ranges. On all plots, JM101 and PB25 operating regions are shown by a *white zone *and a *thick black line*, respectively. **(A) **Maximum plasmid yield. Plasmid-producing results fall into two regions, Planes A and B, whose characteristics are discussed in the text. **(B) **Bla marker production. **(C) **HMP:glycolysis flux ratio. **(D) **Ppc flux as % glucose uptake. **(E) **Mez flux as % glucose uptake. **(F) **Mdh flux as % glucose uptake, where the *white line *is the locus of solutions where Mdh flux = 0.

In practice, wild-type *E. coli *produces acidic by-products (usually acetate) [31], and its Pyk flux is nonzero due to the presence of two Pyk isozymes [32]. Thus, achieving flux distributions like the ones shown in Fig. 2 would be unlikely without using metabolic engineering. To benchmark the wild-type and thus the gain provided by metabolic engineering, the model can be used to predict how maximum plasmid yield varies as a function of *both *acetate and Pyk fluxes. To pursue this sensitivity and benchmarking analysis, the following two constraints were added to the model:

(8)

(9)

where *β *and *φ *are constants that relate glucose uptake to acetate flux and Pyk flux, respectively. Whereas in all previously-discussed models these two fluxes were not constrained, the addition of Equations (8) and (9) enables them to be constrained for any (*β, φ*) combination that results in a feasible solution. Figure 4A shows how maximum plasmid yield varies over feasible (*β, φ*) combinations (i.e. feasible combinations of *r*_*acetate *_and *r*_*Pyk*_) when *α *= 250. Figure 4B shows the concomitant Bla production, and Figures 4C–F show how other key fluxes vary to help permit such plasmid and Bla production.

According to Fig. 4A, the greatest achievable yield (112 mg/g) arises when both *r*_*acetate *_and *r*_*Pyk *_are nil, which is in agreement with earlier results (Fig. 2, *middle*) where the model adopted values of zero for these fluxes when they were left unconstrained. For any (*r*_*acetate*_, *r*_*Pyk*_) combination where at least one of the fluxes is constrained to be nonzero, the yield decreases from the 112 mg/g value. In fact, the yield results fall into two regions, indicated by Planes A and B (Fig. 4A). In each regime, the yield decreases as either of the two fluxes are increased. For the (*r*_*acetate*_, *r*_*Pyk*_) pairs that make up Plane A, this decrease in yield is much steeper when *r*_*Pyk *_is increased for fixed *r*_*acetate *_than when *r*_*acetate *_is increased for fixed *r*_*Pyk*_. In fact, the magnitude of the slope in the former case is more than five-times that in the latter, indicating that Pyk exhibits the more dominant effect in this regime. The opposite is true for the (*r*_*acetate*_, *r*_*Pyk*_) pairs that make up Plane B. There, increasing *r*_*acetate *_for fixed *r*_*Pyk *_causes a sharp decrease in yield, whereas yield remains nearly constant when *r*_*Pyk *_is increased for fixed *r*_*acetate*_. The differing trends in Planes A and B arise from how Ppc, malate dehydrogenase (Mdh), and Mez are used in each (Fig. 4D–F). For all flux distributions in Plane A, Mdh is never operated in the reverse direction (i.e. *r*_*Mdh *_> 0), and Mez is always inactive (i.e. *r*_*Mez *_= 0). In the Plane B distributions, the reverse is true – Mdh is always operated in reverse, and Mez is always active. Here, *r*_*Pyk *_is not high enough to provide the necessary pyruvate for meeting the specified *r*_*acetate*_. Thus, additional pyruvate is created by a cycle that uses increased *r*_*Ppc*_, reverse *r*_*Mdh*_, and nonzero *r*_*Mez*_. On the edge shared by Planes A and B, *r*_*Pyk *_is exactly high enough such that acetate, plasmid, and marker are produced with nil flux through Mez (Fig. 4E) and Mdh (Fig. 4F). In practice, Mez flux is small, if present at all, and Mdh is not operated in reverse during aerobic growth on glucose [20]. If the Ppc-Mdh-Mez cycle were prevented from operating, either by constraining *r*_*Mdh *_> 0 or *r*_*Mez *_= 0, then the model would be infeasible for the (*r*_*acetate*_, *r*_*Pyk*_) pairs that constitute Plane B, leaving only Plane A.

### Yield Horizons of Mutant and Wild-type *E. coli *JM101

The preceding results indicate that the highest plasmid yields are attained when the Pyk and acetate fluxes are zero (Fig. 2 and Fig. 4A). Mutants exist that approach such attributes. In this section, the yield horizons of one mutant and its wild-type are determined, and then contrasted to each other and the best maximal yields.

An *E. coli *mutant that has the activity of the two Pyk isozymes abolished has been constructed and denoted as strain PB25 [33]. Many of the characteristics of PB25 and its wild-type parent JM101 have been well established [19,20,33-37]. Several reports indicate that PB25 produces considerably less acetate than JM101 during batch growth in glucose minimal medium. At high glucose (10 g/L), Zhu et al. [19] found that acetate production occurs with a stoichiometry of *β *= 0.25 and 0.75 for PB25 and JM101, respectively. In terms of a carbon balance, this means that JM101 wastes 25% of input carbon as acetate, whereas PB25 wastes only 8%. At 2 g/L glucose, Ponce [34] reported *β *= 0.33 for PB25 and 0.46 for JM101. Additionally, Flores et al. [20] data indicate that *β *= 0.55 for JM101 when grown on 3 g/L glucose.

Concerning Pyk, *φ *= 0 for PB25, as its Pyk activity has been eliminated [33]. For JM101, Flores et al. [20] measured *φ *= 0.41. Also, Emmerling et al. [36] found *φ *= 0.19 for JM101 grown in a chemostat operated at a dilution rate of 0.40 h^-1 ^with 3.6 g/L glucose.

Based on these reported experimental values, approximate (*r*_*acetate*_, *r*_*Pyk*_) flux regimes for each strain have been highlighted in the results of Fig. 4 (*α *= 250). As this sensitivity analysis shows, even if *r*_*acetate *_and *r*_*Pyk *_are allowed to vary somewhat, the deletion of the two Pyk isozymes in PB25 permits it to attain substantially higher plasmid yields than JM101 (Fig. 4A). PB25 accomplishes this by changing the cell's stoichiometry such that it is better-positioned for plasmid production in three ways: (i) a large *r*_*HMP*_/*r*_*glycolysis *_flux ratio (Fig. 4C), (ii) low acetate production, and (iii) a higher Ppc flux, with no Mez nor reverse Mdh activity needed (Fig. 4D–F).

**Figure 5:**
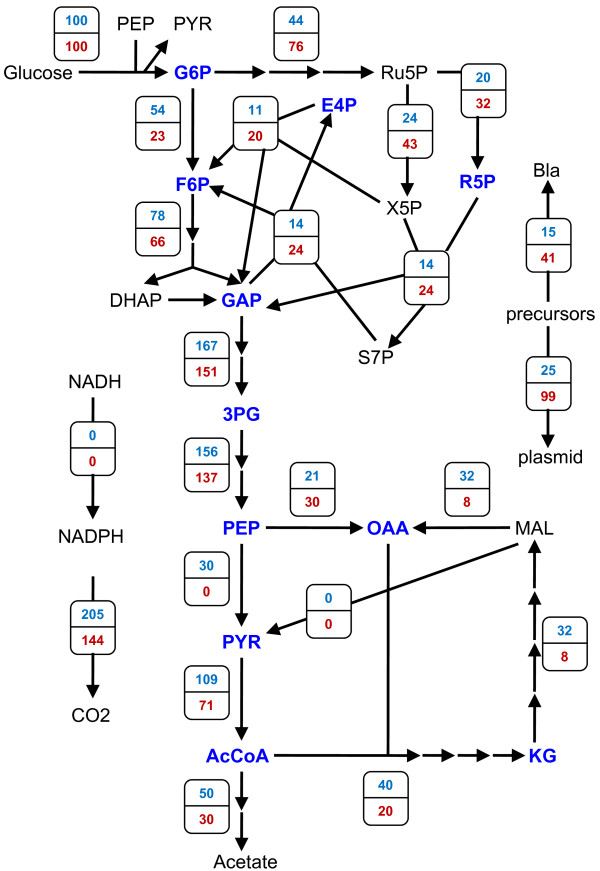
**Maximum plasmid-producing flux distributions for the JM101 and PB25 models**. For both distributions, *α *= 250 in Equation (5), and *r*_*transhydrogenase *_was constrained to zero. Equations (8) and (9) were employed to constrain *r*_*acetate *_and *r*_*Pyk *_to experimental values for JM101 (wild-type) and PB25 (*pykF pykA*). *Top*: JM101. *Bottom*: PB25. For simplicity, the network in Fig. 1 has been condensed and fluxes (mmol/g/h) have been expressed as % of glucose uptake, except *r*_*Bla *_(expressed as % of total protein) and *r*_*plasmid *_(expressed as yield in mg/g). The production of lactate and succinate have also been omitted, as these fluxes were nil for all distributions.

Figure 5 shows the contrast in flux distributions between PB25 (*bottom*) and JM101 (*top*) in greater detail. Here, values of (*β*, *φ*) were selected for each strain that fell within their respective experimental ranges. That is, (*β*, *φ*) = (0.3, 0) for PB25 and (0.5, 0.3) for JM101, hereafter referred to as the *PB25 model *and the *JM101 model*, respectively. In addition, the earlier version of the model, where the acetate and Pyk fluxes were left unconstrained, will be referred to as the *unconstrained model*. As shown in Fig. 5, compared to JM101, PB25 produces 4-fold more plasmid (99 vs. 25 mg/g) and concomitantly 2.7-fold more Bla marker (41 vs. 15% total protein). PB25 does this, in part, by maintaining a higher *r*_*HMP*_/*r*_*glycolysis *_flux ratio (3.3 vs. 0.81) (see also Fig. 3E). Furthermore, PB25 wastes 40% less carbon as acetate, and it also wastes less in the form of CO_2 _despite the higher HMP flux (205 vs. 144). The higher CO_2 _levels of JM101 arise partially due less Ppc flux (30 vs. 21) (see also Fig. 3F) but predominantly due to higher pyruvate dehydrogenase (109 vs. 71) and TCA cycle (40 vs. 20) fluxes, which are brought about by its considerable Pyk activity. Overall, the PB25 model's flux distribution more closely matches that of the highest plasmid-producing unconstrained model (Fig. 2, *middle*).

To contrast the models at other levels of marker expression (i.e. besides *α *= 250), the PB25 and JM101 models can be solved at various values of *α*, as was done already for the unconstrained model (Fig. 3A, E, F). Figures 3B and 3C show how maximum plasmid yield varies with *α *for the JM101 and PB25 models, respectively. For every *α*, the low acetate and nil Pyk phenotype of PB25 endows it with a substantial advantage in terms of plasmid production as compared to the wild-type by enabling an *r*_*HMP*_/*r*_*glycolysis *_flux ratio (Fig. 3E) and Ppc flux (Fig. 3F) that are closer to the unconstrained model. When no marker is produced (*α *= 0), the maximum capacity of the mutant is about 4-fold greater (443 vs. 110 mg/g) than the wild-type. When Bla production is limited, the advantage of PB25 over JM101 widens further. For example, when (*f *= 0.20, α* = 28) in Equation (7) for the PB25 model, the maximum yield is 318 mg/g for all *α *≥ 28. In contrast, the JM101 model is unable to produce Bla at 20% of total protein even at infinite *α*. In fact, even when *α *= 1000 (roughly half that of fully active LacZ production [21,22]), plasmid and marker production are 7.4 mg/g and 17.1% of total protein, respectively. Interestingly, these wild-type values for yield and marker production (Fig. 3C) are on par with those that have been observed experimentally. For example, Rozkov et al. [28] determined that the kanamycin resistance marker is present at 18% of total protein while the pUC-type plasmid yield was 4.9 mg/g.

### Effects of Transhydrogenase Activity

In all models considered thus far, the production of NADPH for biosynthetic reducing power has occurred via (i) two reactions in the oxidative HMP pathway, (ii) one reaction in the TCA cycle, and (iii) a small additional contribution if there is flux through the NADP-dependent Mez (Fig. 1, *red font*). These reactions must generate enough NADPH to meet not only that required for basal biomass demand, but also that required for plasmid and marker synthesis. As a result, the NADPH constraint (see Methods) has a significant impact on the resulting flux distributions. Indeed, a characteristic of high plasmid-producing solutions is high HMP flux (Fig. 4C).

In practice, NADPH may also be supplied via a nicotinamide nucleotide transhydrogenase, which catalyzes the interconversion of NADH and NADPH (Fig. 1). Such interconversion would relax somewhat the constraint that NADPH production imposes on the network. *E. coli *possesses two transhydrogenases, PntAB and UdhA. PntAB is membrane-bound, translocates a proton, and operates in the direction that reduces NADP^+ ^from NADH, whereas UdhA is soluble, energy-independent, and operates in the direction that oxidizes NADPH to form NADH [38]. It has been estimated that PntAB contributes 35–45% of the NADPH needed for biomass synthesis during aerobic batch growth with glucose, whereas UdhA is only needed under conditions of excess NADPH [38].

In each model thus far, transhydrogenase activity has been constrained to be inactive. Therefore, to examine its effect on plasmid production, this constraint was relaxed and given an upper bound set to 40% of NADPH biomass demand, based on the experimental value of Sauer et al. [38]:

(10)

This relaxed transhydrogenase constraint was incorporated into the PB25 and JM101 models, as well as into the unconstrained model. For all *α *in each model, gains in plasmid production resulted when the transhydrogenase was allowed to be active (Fig. 3D). In the unconstrained model, plasmid production increased by 18% at each *α *when the transhydrogenase was active. In the PB25 model, the gain improved to 20% for each *α*. For JM101, the effect of an active transhydrogenase is even greater, with gains of over 82% resulting. When no resources are lost to marker production (i.e. *α *= 0), specific plasmid yield reaches 200 and 533 mg/g in the JM101 and PB25 models, respectively. In the unconstrained model, it reaches 592 mg/g, which is the highest value found in this analysis.

**Figure 6:**
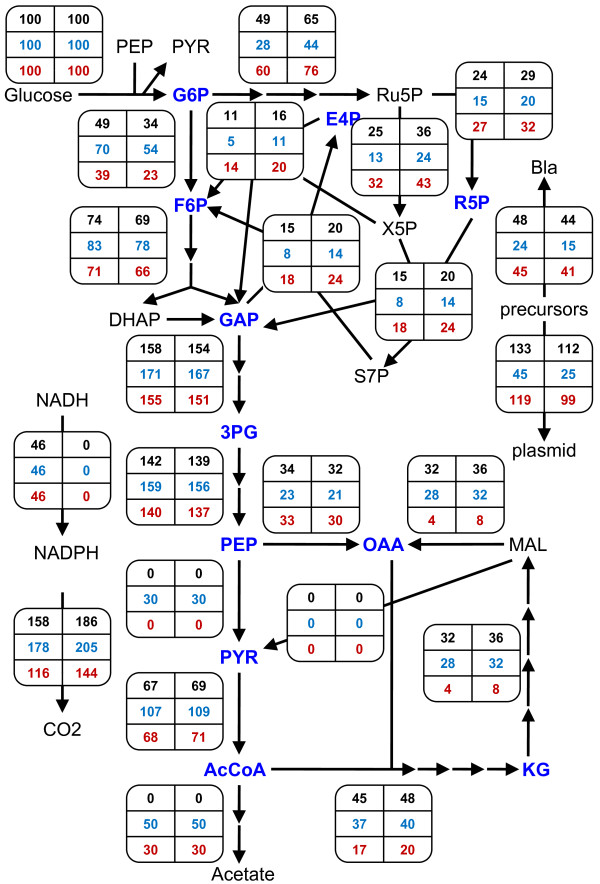
**Effects of transhydrogenase activity on maximum plasmid-producing flux distributions**. For all distributions, *α *= 250 in Equation (5). *Left column*:*r*_*transhydrogenase *_limited to 40% of NADPH biomass demand as per Equation (10). *Right column*:*r*_*transhydrogenase *_constrained to zero; these distributions were copied from Fig. 2 and Fig. 5 to facilitate comparisons. In Equations (8) and (9), *r*_*acetate *_and *r*_*Pyk *_were: unconstrained (*top row*), set to wild-type JM101 values (*middle row*), or set to Pyk-deficient PB25 values (*bottom row*). For simplicity, the network in Fig. 1 has been condensed and fluxes (mmol/g/h) have been expressed as % of glucose uptake, except *r*_*Bla *_(expressed as % of total protein) and *r*_*plasmid *_(expressed as yield in mg/g). The production of lactate and succinate have also been omitted, as these fluxes were nil for all distributions.

How transhydrogenase activity improves plasmid yield can be gleaned from contrasting the flux distributions for the PB25, JM101, and unconstrained cases. These are shown in Figure 6 (*left column*) for *α *= 250 alongside their respective distributions that resulted when the transhydrogenase was constrained to be inactive (*right column*; copied from Fig. 2 and Fig. 5). While the JM101 model showed the greatest percent improvement to plasmid production, as mentioned previously, the absolute gains in plasmid yield were about the same in all three models at roughly 20 mg/g. Gains in plasmid and concomitant marker production necessitate increased precursor usage, leaving less carbon to be given off as CO_2 _and requiring more ATP and NADPH. Because a significant amount of NADPH is produced by the transhydrogenase in these models (i.e. the upper bound is hit in each model), less NADPH needs to be produced elsewhere. Consequently, lower fluxes should be expected through the oxidative HMP pathway and/or isocitrate dehydrogenase. Indeed, while each model shows about the same flux through isocitrate dehydrogenase relative to their respective inactive transhydrogenase case, each exhibits significantly decreased oxidative HMP pathway usage (see also Fig. 3E for additional *α*). This allows a significantly greater fraction of input carbon to directly enter glycolysis, with the ultimate effect being a greater supply of precursor metabolites downstream of GAP that can be directed to plasmid and Bla synthesis. Moreover, decreased oxidative HMP flux, increased Ppc flux (see also Fig. 3F for additional *α*), and less-than-or-equal-to pyruvate dehydrogenase and TCA fluxes collectively mean that less carbon is lost as CO_2_, which further favors plasmid and Bla production.

## Discussion

Through flux analysis, the capacity of *E. coli *for producing plasmid was investigated. Acetate production and the constitutive expression of the plasmid's antibiotic resistance marker were found to exert negative effects, while low Pyk flux and the generation of NADPH by transhydrogenase activity enhance yield. The highest theoretical yield (592 mg/g) resulted under conditions of no marker or acetate production, nil Pyk flux, and the maximum allowable transhydrogenase activity (Fig. 3D, *dashed black line*at *α *= 0). Such a yield is 12-fold higher than the best reported value [8], which suggests that strain engineering can be done to potentially elevate yield further. The lowest potential yields resulted when Pyk and acetate fluxes were constrained to wild-type levels (Fig. 3B and Fig. 4A). Further, these predicted levels of yield (on the order of tens of mg/g) and concomitant marker production (10–18% of total protein) are comparable to those reported experimentally [28].

These results and conclusions, however, should be considered in terms of the limitations of flux models and contrasted further to existing experimental results. When one conservatively views the predictions of flux models, they can be viewed as indicating the best a cell can do given (i) the constraints imposed, (ii) the nature of the nested metabolic reactions, and (iii) the competing demands on resources that occur given that some beneficial redundancy exists, such as multiple points for NADPH generation. Regulation and other limitations are typically not considered. In this case, one major assumption was that plasmid replication was constrained more by precursor supply than by the negative control over replication frequency or DNA polymerase activity.

We suggest that such a limitation does not necessarily negate the value of predictions from flux models. Rather, if the aim is to alter cells through extensive metabolic engineering, then it seems useful to have the best yield and target flux distribution identified ahead of time. Knowing whether current yields already approach the theoretical yield is worthwhile because it indicates that research resources can be better directed elsewhere. On the other hand, when the yield horizon is promising, the optimal flux distribution provides the ultimate target for the mutagenesis tasks.

Prior work also suggests that informative and implementable results are often attained. One example is provided by the metabolic engineering of *B. subtilis *for the purpose of producing more folic acid. Flux analysis predicted that suppressing Pyk activity was amongst the attributes of an optimal flux distribution. Subsequent experimentation determined that this mutation, in conjunction with others, increased the folic acid titer by 700% [15].

To scrutinize further the predictions reported here, a comparison to prior work on *E. coli *expressing high copy number plasmids can be made. Here, how adaptations compare to the trends in the optimal flux distributions can provide an indication of the reasonableness of the optimal trends. In one study, *E. coli *JM101 grown in M9 minimal medium containing glucose (i.e. the same strain and growth conditions modeled in this study) exhibited less growth rate burden from plasmid maintenance when the HMP pathway gateway enzyme, glucose-6-phosphate dehydrogenase (Zwf) was over-expressed [39]. Transformation with a multicopy plasmid resulted in a growth rate decrease from 0.70 to 0.46 h^-1^; the growth rate recovered to 0.57 and 0.64 h^-1^, however, when Zwf activity increased 9- and 12-fold, respectively [39]. In another study, Ow et al. [40] found that when *E. coli *DH5α harbored a high copy plasmid, expression from *zwf *increased by 1.2-fold, while expression from phosphoglucose isomerase decreased by 1.8-fold. Provided that flux correlates with expression level, their results suggest that one adaptation to plasmid presence entails increasing the *r*_*HMP*_/*r*_*glycolysis *_ratio by about a factor of 2. Lastly, Birnbaum and Bailey [41] reported that *E. coli *contained significantly less Pyk and significantly more Ppc when copy number increased.

The optimal *r*_*HMP*_/*r*_*glycolysis *_ratio, in turn, was predicted to range from roughly 1 to 2, where the particular value depends on the transhydrogenase activity (Fig. 3E, *black lines*). This optimal value of the ratio is 3.5- to 7-fold greater than the experimentally observed wild-type JM101 value of 0.29 [20]. In the optimal solutions, the Ppc flux is much greater than that of Pyk regardless of transhydrogenase activity (Fig. 3F, *black lines*). Indeed, the best plasmid-producing solutions show nil Pyk activity. In contrast, Ppc flux has been measured to be only 60% that of Pyk for wild-type JM101 [20], a feature which was also captured reasonably well by the JM101 model (Fig. 5, *top*; 70%). Overall, the trends in the optimal flux solutions thus both track and combine the HMP/glycolysis, Pyk, Ppc, and other flux adaptations reported to occur when high copy number plasmids are maintained.

Thus, the match between the trends in the optimal flux distributions and experimental results raises the prospect that the particular flux values can serve as useful guideposts. Furthermore, suppressing Pyk activity appears to be a key facet of the optimal solutions. Abolishing Pyk provides a hard constraint that alters cellular stoichiometry such that Ppc and HMP fluxes are forced to increase, while acetate formation is suppressed. These changes provide improved precursor supply for plasmid and concomitant marker synthesis, as well as ensure that the necessary NADPH production occurs. These features also can be viewed as "bundling" the individually observed adaptations described above for maintaining high copy number plasmids.

To experimentally explore the prediction that abolishing Pyk activity leads to flux changes that can increase plasmid yield, we recently compared and reported the plasmid copy numbers attained by JM101 and PB25 [16]. These copy numbers were obtained for pGFPuv (i.e. the same model plasmid used in this theoretical analysis) during exponential growth in glucose minimal medium. Because pGFPuv employs a temperature-sensitive pUC *ori*, the copy numbers were measured in cells growing isothermally at 37°C as well as in cells that were shifted from 37 to 42°C, where copy number typically increases 2- to 3-fold over several generations [2,5,7,10,11], as mentioned previously.

When grown at 37°C, PB25 maintained a 4-fold higher copy number than JM101. After being temperature-shifted, PB25 contained over 9-fold more plasmid than isothermally-grown JM101. Furthermore, when the strains are compared on a temperature-shifted basis, the copy number attained by JM101 was less than half that attained by PB25, whose copy number at 42°C reached over 1200. Overall, PB25 was found to contain considerably more plasmid than JM101, while PB25 maintained 78 and 92% of the specific growth rate of JM101 at 37 and 42°C, respectively. The comparable growth rates indicate that growth rate changes alone cannot account for the significantly different plasmid contents. Moreover, after the temperature shift, new plateau levels of plasmid/cell were attained after about three generations, indicating that balanced growth was achieved by each strain and that each exhibited significantly different rates of plasmid synthesis.

Based on the modeling results, we interpret the experimental results described above as follows. Increasing temperature increased the copy number in both JM101 and PB25 to new plateaus; hence, negative control over plasmid replication remains a factor in pUC-type plasmids. However, when grown at 42°C, PB25 maintained a growth rate similar to JM101 despite possessing much more plasmid and comparable or more marker synthesis. Thus, as suggested by the modeling, PB25 possesses a flux distribution that enables extra DNA and marker synthesis to be better tolerated while significant cell growth still occurs. These conclusions then suggest that diminishing negative control further in a metabolic background provided by PB25 may further increase the copy number. An approach such as that described by Tomizawa [42] could potentially be used to introduce additional *ori *mutations that might further weaken negative control beyond the level already provided by the *rop *and pUC mutations, bearing in mind that additional non-*ori *sequence-specific effects may also prove beneficial [8]. Additionally, yield enhancements might result with the over-expression or deletion of other key proteins involved in plasmid replication. However, Williams et al. [8] have demonstrated that this is not the case after examining a number of such replication factors. Finally, maintaining a similar growth rate despite increased plasmid content suggests that DNA polymerase activity does not yet present a limit. That is, if total DNA polymerase activity was significantly taxed, one would expect that the time required for chromosomal DNA replication, and thus the cell cycle time, would have lengthened considerably.

## Conclusion

The capacity of *E. coli *for producing plasmid DNA was examined using metabolic flux analysis, and factors were identified that significantly influence specific yield. Both the production of acetate and the plasmid-encoded antibiotic resistance marker negatively impact plasmid production, whereas transhydrogenase activity and low/nil Pyk flux offer positive effects. The highest theoretical yield (592 mg/g) resulted under conditions of no acetate or marker production, nil Pyk flux, and maximum transhydrogenase activity. As this yield is 12-fold higher than the best reported to date, it implies that metabolic engineering of the host strain background might aid in narrowing the gap between the theoretical yield and the achievable yield. One model-based mutation, the deletion of Pyk, has been explored experimentally. In glucose minimal media, it produced substantially more plasmid DNA than the wild-type with only a modest penalty to growth rate, thus corroborating the model predictions.

Additional strain engineering strategies suggested by the model include transhydrogenase up-regulation and minimization of marker expression, or perhaps a shift away from selection via antibiotic resistance all together. A precedent has already been established in the former case, where transhydrogenase over-expression has helped to increase the productivity and yield of poly(3-hydroxybutyrate) [43]. Weakening marker expression or using an alternative means of selection have also been explored by other groups, mainly to avoid patient safety issues associated with the presence of the marker in the final formulation [44]. Here, we have demonstrated theoretically that minimization/elimination of marker synthesis also offers the added benefit of freeing up resources that can be channelled into additional plasmid output.

## Appendix

### Estimation of Maximum Theoretical Plasmid Yield via Simple Carbon Balance

For the case of growth on minimal glucose medium, carbon enters the cell as either glucose or CO_2_. This carbon is used to produce biomass, and it is also given off as CO_2 _and excreted as acid by-products (e.g. acetate, lactate, succinate). When replicating a plasmid and constitutively producing its antibiotic resistance marker, carbon must also be used for these two species. This gives the following overall carbon balance reaction:

(A1)

where CO_2 _is located on the right-hand side to reflect its net production.

The amount of input carbon (mmol carbon/g) from glucose equals 95.2 based on its molecular weight, the fact that there are six carbons per glucose, and that 0.35 g/g glucose is a typical biomass yield for aerobic growth in glucose minimal medium [19]. The amount of carbon in biomass is 43.2, which was calculated based on the required amounts of precursor metabolites [17] and their respective amounts of carbon per metabolite. This consumes approximately 45% of the input carbon. If we assume that, say, 30% is given off as CO_2_, this leaves 25%. If we further assume (i) that all of this remaining carbon is put toward plasmid production, instead of lost as acid by-products, and (ii) that we neglect marker production, then the maximum attainable plasmid yield for a model plasmid, pGFPuv, is 742 mg/g. This number was arrived at using the chemical formula of pGFPuv (C_65131_H_75142_O_40044_N_24968_P_6674_) and its corresponding molecular weight. For the 3337-bp pGPFuv plasmid, this yield corresponds to roughly 33,300 copies/cell. This plasmid was chosen because it is pUC-based, commercially available, and contains a *gfp*_*uv *_gene that acts as a dummy therapeutic gene sequence.

## Abbreviations

αKG: α-ketoglutarate; μ: specific growth rate (h^-1^); 2PG: 2-phosphoglycerate; 3PG: 3-phosphoglycerate; 6PG: 6-phosphogluconate; 6PGL: 6-phosphogluconolactone; AcCoA: acetyl-coenzyme A; Ac-P: acetyl-phosphate; Bla: β-lactamase; BPG: 1,3-bisphosphoglycerate; CIT: citrate; dATP: deoxyadenosine triphosphate; dCTP: deoxycytosine triphosphate; dGTP: deoxyguanosine triphosphate; DHAP: dihydroxyacetone phosphate; dNTP: deoxyribonucleoside triphosphate; dTTP: deoxythymidine triphosphate; F6P: fructose-6-phosphate; FBP: fructose-1,6-bisphosphate; FUM: fumarate; G6P: glucose-6-phosphate; GAP: glyceraldehyde phosphate; HMP: hexose monophosphate pathway; IC: isocitrate; LacZ: β-galactosidase; MAL: malate; Mdh: malate dehydrogenase; Mez: malic enzyme(s); OAA: oxaloacetate; *ori*: origin of replication; PEP: phosphoenolpyruvate; Ppc: phosphoenolpyruvate carboxylase; PTS: phosphotransferase system; Pyk: pyruvate kinase; PYR: pyruvate; R5P: ribose-5-phosphate; r_*i*_: flux of *i *(e.g. r_Pyk _is the flux of pyruvate kinase); Ru5P: ribulose-5-phosphate; S7P: sedoheptulose-7-phosphate; SUC: succinate; Succ-CoA: succinyl-coenzyme A; TCA: tricarboxylic acid cycle; X5P: xylulose-5-phosphate.

## Competing interests

The authors declare that they have no competing interests.

## Authors' contributions

DSC performed the experiments and carried out the modeling (design, assembly, results compilation), as well as participated in the interpretation of the results and the drafting of the manuscript. RRK provided advice on the experiments and participated in the interpretation of the results and editing of the manuscript. MMA was involved in the modeling design and participated in the interpretation of the results and editing of the manuscript. MMD conceived the study, oversaw the modeling, and participated in the interpretation of the results and the drafting of the manuscript. All authors read and approved the final manuscript.

## References

[B1] Gene Therapy Clinical Trials Worldwide. http://www.wiley.co.uk/genetherapy/clinical/.

[B2] Lahijani R, Hulley G, Soriano G, Horn NA, Marquet M (1996). High-yield production of pBR322-derived plasmids intended for human gene therapy by employing a temperature-controllable point mutation. Hum Gene Ther.

[B3] O'Kennedy RD, Ward JM, Keshavarz-Moore E (2003). Effects of fermentation strategy on the characteristics of plasmid DNA production. Biotechnol Appl Biochem.

[B4] Wang Z, Le G, Shi Y, Wegrzyn G (2001). Medium design for plasmid DNA production based on stoichiometric model. Process Biochem.

[B5] Carnes AE, Hodgson CP, Williams JA (2006). Inducible *Escherichia coli *fermentation for increased plasmid DNA production. Biotechnol Appl Biochem.

[B6] Danquah MK, Forde GM (2007). Growth medium selection and its economic impact on plasmid DNA production. J Biosci Bioeng.

[B7] Danquah M, Forde G (2008). Development of a pilot-scale bacterial fermentation for plasmid-based biopharmaceutical production using a stoichiometric medium. Biotechnol Bioprocess Eng.

[B8] Williams JA, Luke J, Langtry S, Anderson S, Hodgson CP, Carnes AE (2009). Generic plasmid DNA production platform incorporating low metabolic burden seed-stock and fed-batch fermentation processes. Biotechnol Bioeng.

[B9] Lin-Chao S, Bremer H (1986). Effect of the bacterial growth rate on replication control of plasmid pBR322 in *Escherichia coli*. Mol Gen Genet.

[B10] Lin-Chao S, Chen WT, Wong TT (1992). High copy number of the pUC plasmid results from a Rom/Rop-suppressible point mutation in RNA II. Mol Microbiol.

[B11] Miki T, Yasukochi T, Nagatani H, Furuno M, Orita T, Yamada H, Imoto T, Horiuchi T (1987). Construction of a plasmid vector for the regulatable high level expression of eukaryotic genes in *Escherichia coli*: an application to overproduction of chicken lysozyme. Protein Eng.

[B12] (1998). Guidance for Industry: Guidance for Human Somatic Cell Therapy and Gene Therapy.

[B13] (2007). Guidance for Industry: Considerations for Plasmid DNA Vaccines for Infectious Disease Indications.

[B14] Varma A, Boesch BW, Palsson BO (1993). Biochemical production capabilities of *Escherichia coli*. Biotechnol Bioeng.

[B15] Zhu T, Pan Z, Domagalski N, Koepsel R, Ataai MM, Domach MM (2005). Engineering of *Bacillus subtilis *for enhanced total synthesis of folic acid. Appl Environ Microbiol.

[B16] Cunningham DS, Liu Z, Domagalski N, Koepsel RR, Ataai MM, Domach MM (2009). Pyruvate kinase-deficient *Escherichia coli *exhibits increased plasmid copy number and cyclic AMP levels. J Bacteriol.

[B17] Neidhardt FC, Ingraham JL, Schaechter M (1990). Physiology of the bacterial cell: A molecular approach.

[B18] Neidhardt FC, Curtiss R, Ingraham JL, Lin ECC, Low KB, Magasanik B, Reznikoff WS, Riley M, Schaechter M, Umbarger HE (1996). *Escherichia coli *and *Salmonella*: Cellular and molecular biology.

[B19] Zhu T, Phalakornkule C, Koepsel RR, Domach MM, Ataai MM (2001). Cell growth and by-product formation in a pyruvate kinase mutant of *E. coli*. Biotechnol Prog.

[B20] Flores S, Gosset G, Flores N, de Graaf AA, Bolivar F (2002). Analysis of carbon metabolism in *Escherichia coli *strains with an inactive phosphotransferase system by ^13^C labeling and NMR spectroscopy. Metab Eng.

[B21] Kierzek AM, Zaim J, Zielenkiewicz P (2001). The effect of transcription and translation initiation frequencies on the stochastic fluctuations in prokaryotic gene expression. J Biol Chem.

[B22] Kennell D, Riezman H (1977). Transcription and translation initiation frequencies of the *Escherichia coli lac *operon. J Mol Biol.

[B23] Lee S, Phalakornkule C, Domach MM, Grossmann IE (2000). Recursive MILP model for finding all the alternate optima in LP models for metabolic networks. Comput Chem Eng.

[B24] Soubrier F, Cameron B, Manse B, Somarriba S, Dubertret C, Jaslin G, Jung G, Caer CL, Dang D, Mouvault JM (1999). pCOR: a new design of plasmid vectors for nonviral gene therapy. Gene Ther.

[B25] Cranenburgh RM, Hanak JA, Williams SG, Sherratt DJ (2001). Escherichia coli strains that allow antibiotic-free plasmid selection and maintenance by repressor titration. Nucleic Acids Res.

[B26] Hagg P, de Pohl JW, Abdulkarim F, Isaksson LA (2004). A host/plasmid system that is not dependent on antibiotics and antibiotic resistance genes for stable plasmid maintenance in *Escherichia coli*. J Biotechnol.

[B27] Panayotatos N (1988). Recombinant protein production with minimal-antibiotic-resistance vectors. Gene.

[B28] Rozkov A, Avignone-Rossa CA, Ertl PF, Jones P, O'Kennedy RD, Smith JJ, Dale JW, Bushell ME (2004). Characterization of the metabolic burden on *Escherichia coli *DH1 cells imposed by the presence of a plasmid containing a gene therapy sequence. Biotechnol Bioeng.

[B29] Bremer H, Dennis PP, Neidhardt FC, Curtiss R III, Ingraham JL, Lin ECC, Low KB, Magasanik B, Reznikoff WS, Riley M, Schaechter M, Umbarger HE (1996). Modulation of chemical composition and other parameters of the cell by growth rate. Escherichia coli and Salmonella: Cellular and molecular biology.

[B30] Seo JH, Bailey JE (1985). Effects of recombinant plasmid content on growth properties and cloned gene product formation in *Escherichia coli*. Biotechnol Bioeng.

[B31] Luli GW, Strohl WR (1990). Comparison of growth, acetate production, and acetate inhibition of *Escherichia coli *strains in batch and fed-batch fermentations. Appl Environ Microbiol.

[B32] Malcovati M, Kornberg HL (1969). Two types of pyruvate kinase in *Escherichia coli *K12. Biochim Biophys Acta.

[B33] Ponce E, Flores N, Martinez A, Valle F, Bolivar F (1995). Cloning of the two pyruvate kinase isoenzyme structural genes from *Escherichia coli*: the relative roles of these enzymes in pyruvate biosynthesis. J Bacteriol.

[B34] Ponce E (1999). Effect of growth rate reduction and genetic modifications on acetate accumulation and biomass yields in *Escherichia coli*. J Biosci Bioeng.

[B35] Ponce E, Martinez A, Bolivar F, Valle F (1998). Stimulation of glucose catabolism through the pentose pathway by the absence of the two pyruvate kinase isoenzymes in *Escherichia coli*. Biotechnol Bioeng.

[B36] Emmerling M, Dauner M, Ponti A, Fiaux J, Hochuli M, Szyperski T, Wuthrich K, Bailey JE, Sauer U (2002). Metabolic flux responses to pyruvate kinase knockout in *Escherichia coli*. J Bacteriol.

[B37] Siddiquee KAZ, Arauzo-Bravo MJ, Shimizu K (2004). Metabolic flux analysis of *pykF *gene knockout *Escherichia coli *based on ^13^C-labeling experiments together with measurements of enzyme activities and intracellular metabolite concentrations. Appl Microbiol Biotechnol.

[B38] Sauer U, Canonaco F, Heri S, Perrenoud A, Fischer E (2004). The soluble and membrane-bound transhydrogenases UdhA and PntAB have divergent functions in NADPH metabolism of *Escherichia coli*. J Biol Chem.

[B39] Flores S, de Anda-Herrera R, Gosset G, Bolivar FG (2004). Growth-rate recovery of *Escherichia coli *cultures carrying a multicopy plasmid, by engineering of the pentose-phosphate pathway. Biotechnol Bioeng.

[B40] Ow DS-W, Nissom PM, Philp R, Oh SK-W, Yap MG-S (2006). Global transcriptional analysis of metabolic burden due to plasmid maintenance in *Escherichia coli *DH5α during batch fermentation. Enzyme Microb Technol.

[B41] Birnbaum S, Bailey JE (1991). Plasmid presence changes the relative levels of many host cell proteins and ribosome components in recombinant *Escherichia coli*. Biotechnol Bioeng.

[B42] Tomizawa J (1984). Control of ColE1 plasmid replication: the process of binding of RNA I to the primer transcript. Cell.

[B43] Sanchez AM, Andrews J, Hussein I, Bennett GN, San KY (2006). Effect of overexpression of a soluble pyridine nucleotide transhydrogenase (UdhA) on the production of poly(3-hydroxybutyrate) in *Escherichia coli*. Biotechnol Prog.

[B44] Mairhofer J, Grabherr R (2008). Rational vector design for efficient non-viral gene delivery: challenges facing the use of plasmid DNA. Mol Biotechnol.

